# Operando Study of the Active Phase in Liquid GaPt Alloy Catalysts

**DOI:** 10.1002/smsc.202500423

**Published:** 2025-11-28

**Authors:** Michael S. Moritz, Christoph Wichmann, Marius Steinmetz, Hans‐Peter Steinrück, Christian Papp

**Affiliations:** ^1^ Lehrstuhl für Physikalische Chemie II Friedrich‐Alexander‐Universität Erlangen‐Nürnberg Egerlandstr. 3 91058 Erlangen Germany; ^2^ Angewandte Physikalische Chemie Freie Universität Berlin Arnimallee 22 14195 Berlin Germany

**Keywords:** active site, catalysis, dehydrogenation, liquid metals, near‐ambient pressure X‐ray photoelectron spectroscopy, operando, quadrupole mass spectrometry

## Abstract

The *operando* study investigates propane dehydrogenation (PDH) using a liquid gallium‐platinum catalyst based on the supported catalytically active liquid metal solutions (SCALMS) concept. The focus is on monitoring and analyzing the active phase during reaction using near‐ambient pressure X‐ray photoelectron spectroscopy (NAPXPS) combined with gas phase analysis. This approach proves practical in tracking surface changes and chemical states during catalytic reactions, providing real‐time insights into the catalyst behavior. PDH, an industrially significant reaction, is investigated using a GaPt SCALMS with 1 at.% Pt content. The findings reveal that metallic liquid GaPt SCALMS exhibit high activity, while the presence of oxygen in the feed stream significantly lowers the activity of the catalyst. While current liquid metal catalysts often experience an activation period, a pathway to achieving stable conversion rates right after the start is demonstrated. This stability lays a foundation for developing next‐generation catalysts with improved performance. The investigation also highlights the critical influence of oxidic Ga on catalytic activity, offering valuable guidance for optimizing catalyst design. Overall, the findings underscore the practical importance of NAPXPS in advancing the understanding of surface properties in catalytic systems.

## Graphical abstract

This operando near‐ambient pressure X‐ray photoelectron spectroscopy study investigates the propane dehydrogenation using a liquid gallium‐platinum catalyst. The catalyst shows high activity and coking resistance of the liquid metal surface, while the presence of oxygen significantly lowers the activity. Without oxygen in the feed stream, the catalyst is reduced and reactivates quickly.

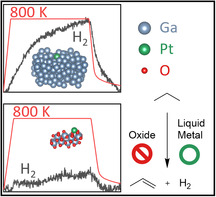

## 1 Introduction

Thanks to catalyst research, industrial chemical processes are becoming more energy‐efficient, resource‐efficient, and profitable.^[^ ^1^ ^]^ Heterogeneous catalyst systems are vital in chemical industry due to their robustness under extreme reaction conditions and ease of product separation from reaction mixtures, lowering operational complexity and costs.^[^ ^2^, ^3^, ^4^ ^]^ Additionally, they are highly scalable, making them ideal candidates for large‐scale industrial applications like petroleum refining and bulk chemical synthesis.

To gain insight into the catalytic mechanisms of these systems, model studies are of para‐mount importance.^[^ ^5^, ^6^, ^7^ ^]^ Model systems for heterogeneous catalysts are typically simplified versions of the real catalyst system. Classical model systems often are idealized surfaces of active materials, like bulk single crystals or nanoparticles on flat supports. They mimic the nanoparticles of active material on porous supports, an established way of using catalytic materials in industry. Herein, we use an extended bulk sample as a first step to gain fundamental knowledge. With these fundamental insights, the interpretation of nanoscale data can become possible. In most model studies, the samples are investigated in a clean and idealized environment, often ultrahigh vacuum (UHV). This simplification, however, alters the system, limiting many model studies in their relevance for actual catalyst systems. In the here‐presented investigation, a near‐ambient pressure setup is used to allow for studies under conditions relevant to the interpretation of reactor studies.^[^ ^8^, ^9^, ^10^ ^]^

Supported catalytically active liquid metal solutions (SCALMS) present a unique approach to heterogeneous catalysis. These systems are composed of alloys containing a low‐melting‐point liquid metal matrix (typically Ga, In, Sn)^[^ ^11^, ^12^ ^]^ and small amounts of catalytically active transition metals to form a liquid alloy at reaction temperatures (typically < 10 at.% of Pt,^[^ ^13^, ^14^, ^15^, ^16^ ^]^ Rh,^[^ ^17^ ^]^ Pd,^[^ ^18^ ^]^ Ni,^[^ ^19^, ^20^ ^]^ or Cu^[^ ^21^ ^]^). As liquid or gaseous organic reactants do not dissolve in Ga, SCALMS generate a heterogeneous phase. They exhibit great activity and feature higher selectivity compared to traditional solid catalytic systems during the dehydrogenation of light alkanes.^[^ ^18^ ^]^ They surpass them in terms of stability and resistance to coking and poisoning.^[^ ^14^, ^22^, ^23^ ^]^ These advantages are due to the site isolation of the dissolved active transition metal atoms and the highly dynamic nature of SCALMS.^[^ ^13^, ^14^, ^17^, ^18^, ^24^, ^25^ ^]^

Herein, we present the first *operando* near‐ambient pressure X‐ray photoelectron spectroscopy (NAPXPS) study on SCALMS, highlighting the impact of surface oxidation on catalytic activity. The significance of these results lies in establishing a new foundation for future SCALMS research, providing timely and highly relevant insights into the nature of the active site and how catalytic feedstock and feedstock contaminations influence SCALMS catalyst performance. Furthermore, this study provides evidence for this promising concept, as the most fundamental and ideal macroscopic model surface of a SCALMS catalyst was shown to be a highly active surface for the dehydrogenation of light alkanes.

## 2 Results and Discussion

### 2.1 Propane Dehydrogenation (PDH): tr‐NAPXPS and Quadrupole Mass Spectrometry (QMS)

The catalytic activity of a macroscopic GaPt alloy droplet with 1 at.% Pt was tested *operando* during a temperature ramp from room temperature to 800 K, while exposed to ≈0.1 mbar (estimated from background pressure reading) of propane. The activity of this SCALMS model system toward PDH was obtained by recording the H_2_ formation with QMS, and the chemical changes on the catalyst surface were assessed by NAPXPS.

**Figure 1:**
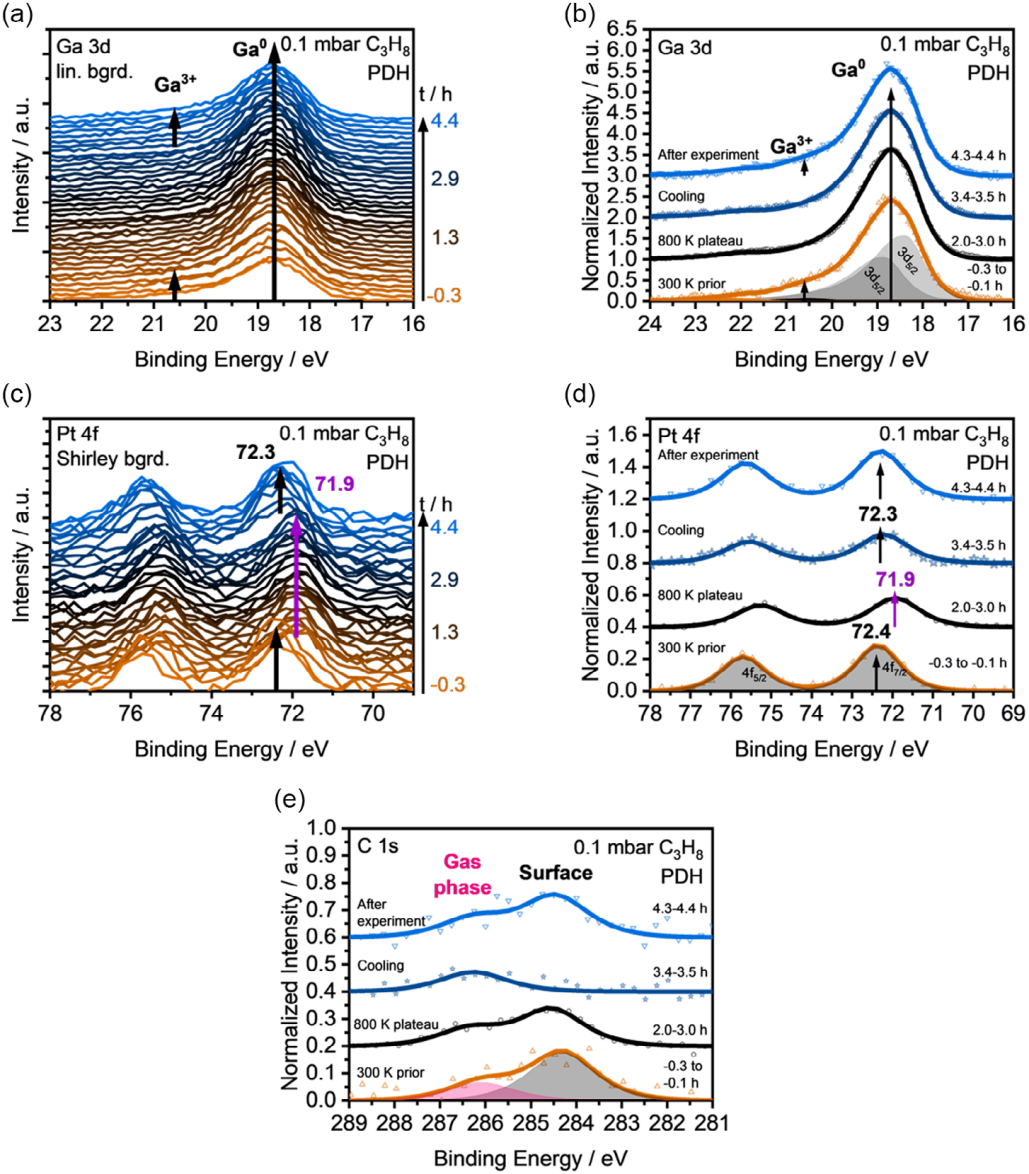
Operando data (0.1 mbar C_3_H_8_) for a 1 at.% Pt in liquid Ga sample: a) XPS of the Ga 3d region; b) averaged Ga 3d spectra (prior to heating: from −0.3 to −0.1 h, 800 K plateau: 2.0–3.0 h, during cooling: 3.4–3.5 h, after heating: 4.3–4.4 h) and with one exemplary fit envelope; c) XPS of the Pt 4f region, and d) shows the averaged Pt 4f regions; and e) averaged C 1s regions.

**Figure 2:**
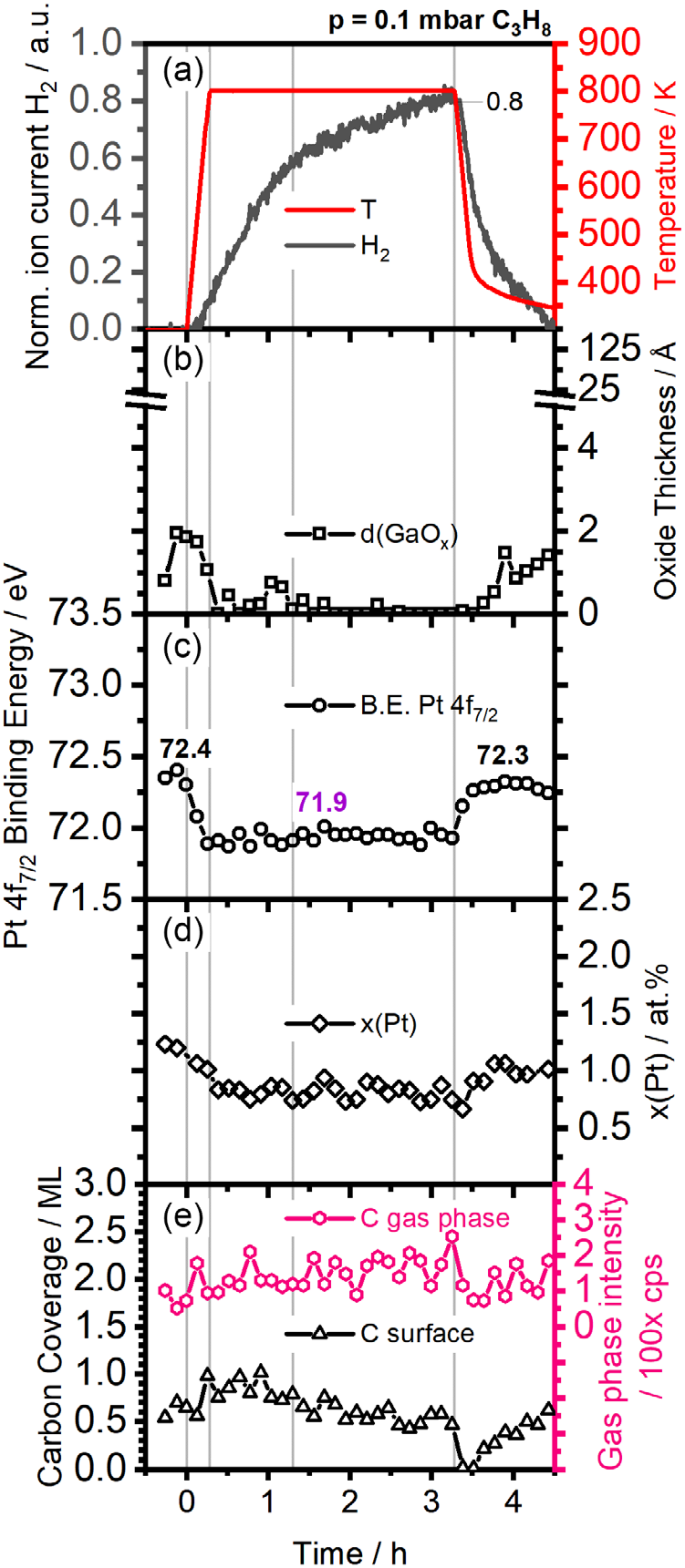
Operando data (0.1 mbar C_3_H_8_) for a 1 at.% Pt in liquid Ga sample: a) activity, b) oxide thickness, c) Pt binding energy, d) Pt concentration in the surface near region, and e) carbon gas phase and surface coverage. Vertical lines are given at 0.0, 0.3, 1.3, and 3.3 h.

The spectroscopic results of this *operando* experiment are shown in **Figure** **1**. First, only fit details will be discussed with Figure 1. A detailed discussion of effects observed during the *operando* experiment is found below in **Figure** **2**.

Figure 1a shows a waterfall plot of the Ga 3d region during the operando experiment. For a more detailed analysis, averaged spectra of specific time frames and a representative peak fit are shown in Figure 1b. The metallic contribution is fitted by spin‐orbit‐split Ga 3d_5/2_ and Ga 3d_3/2_ components at 18.4 and 18.9 eV, respectively (with a splitting of +0.46 eV).^[^ ^26^, ^27^, ^28^, ^29^ ^]^ The shoulder at +2.15 eV to higher binding energy is assigned to a Ga^3+^ contribution and is fitted by Ga 3d_5/2_ and Ga 3d_3/2_ components at 20.6 and 21.1 eV, respectively.^[^ ^30^, ^31^ ^]^ In addition, we observe a weak feature at 22.0 eV, that is, at +3.6 eV relative to the metallic Ga 3d_5/2_ component; it is assigned to a shake‐up feature with 5% intensity.^[^ ^26^, ^32^, ^33^ ^]^ No shifts of the Ga 3d main peak are detected during the experiment. For better visibility of the Ga^3+^ contribution, zoomed spectra are given in Figure S2, Supporting Information.

The Pt 4f spectra collected during the *operando* experiment are shown in Figure 1c as a waterfall plot. Additionally, the averaged spectra of relevant time frames are given in Figure 1d. The signal obtained at 300 K before heating (from −0.3 to −0.1 h) shows a spin‐orbit‐split Pt 4f_7/2_ and 4f_5/2_ doublet, which is fitted by two peaks at 72.4 and 75.7 eV, respectively (with a splitting of +3.33 eV). The Pt 4f_7/2_ peak position is assigned to Pt‐rich intermetallic phases present in or on the solid GaPt alloy.^[^ ^11^, ^34^, ^35^ ^]^ Such solid intermetallic phases were observed previously in nanoscale high‐resolution transmission electron microscopy studies.^[^ ^36^ ^]^ After heating to the reaction temperature of 800 K and after an activation period (2.0–3.0 h), a different species with a lower Pt 4f_7/2_ binding energy is found at 71.9 ± 0.1 eV. Since the Pt‐rich GaPt intermetallic phases in a GaPt alloy with 1.0 at.% Pt melt at ≈530 K,^[^ ^37^ ^]^ we assign this binding energy to Pt atoms in liquid Ga. In literature, similar Pt 4f binding energy shifts are observed at high temperatures in UHV and assigned to isolated Pt in liquid matrices.^[^ ^11^, ^31^ ^]^ Further, Bauer et al. detected isolated Pt atoms at the liquid GaPt surface with *operando* diffuse reflectance infrared Fourier transform spectroscopy (DRIFTS) during the PDH.^[^ ^13^ ^]^ By tracking the temperature‐dependent surface concentration and binding energy of the active transition metals in SCALMS with XPS, and correlating this data with density‐functional theory (DFT), we are able to observe the transition to a liquid metal alloy and identify single transition metal atoms in this liquid.^[^ ^11^, ^17^, ^29^, ^38^, ^39^, ^40^, ^41^ ^]^ Upon cooling (>3.4 h), the Pt peak shifts back to 72.3 ± 0.1 eV. We assign this shift to the reformation of Pt‐rich phases, similar to those present prior to the experiment.

In Figure 1e, the averaged spectra of the C 1s region are shown. The spectrum measured prior to heating (−0.3 and −0.1 h) displays two contributions at 284.3 and 286.1 eV, which are assigned to surface carbon and the C_3_H_8_ gas phase signal, respectively.^[^ ^42^, ^43^, ^44^ ^]^ In UHV, this gas phase signal disappears (Figure S3a, Supporting Information). The C_3_H_8_ gas phase signal without the sample is found at 287.3 eV (Figure S3b, Supporting Information). When the sample is introduced, it shifts to lower binding energies due to changes in the work function.^[^ ^45^ ^]^ During the reaction (2.0–3.0 h), the surface species shifts to + 0.2 eV higher binding energy, that is, 284.5 ± 0.1 eV, and drops in intensity by ≈20%. As the sample is cooled down and the reaction stops, no surface carbon species are observed at ≈285 eV, and only the gas phase signal remains. After the operando experiment (4.3–4.4 h), a gas phase and a surface carbon signal are again observed.

For a detailed discussion of effects observed during the *operando* experiment, reduced spectroscopic data are correlated with the QMS activity data on the same time scale in Figure 2.

Figure 2a shows the temperature ramp (red) and the H_2_ partial pressure resulting from the PDH at a propane pressure of ≈0.1 mbar. Note that the pressure was set to this value already before the start of the experiment with the sample at room temperature, in order to determine the H_2_ background signal. The linear temperature ramp of 30 K min^−1^ starts at t  = 0.0 h. From ≈0.2 h, that is, at ≈620 K, we observe an increase in H_2_ pressure, which is indicative of the dehydrogenation reaction. While after 0.3 h the final temperature of 800 K is reached, the H_2_ signal continues to increase linearly until 1.3 h (0.6 a.u.) and with a lower slope beyond to reach 0.8 a.u. at 3.0 h. This behavior suggests an activation period of the catalyst. After 3.3 h, a cooling ramp of −30 K min^−1^ is started. The H_2_ signal initially remains at 0.8 a.u. until it drops sharply at 3.4 h as the temperature falls below ≈660 K. At 4.50 h, that is, at 345 K, the H_2_ signal has dropped back to the initial background value. We interpret the temperature range ≈620–660 K to include the lowest required temperature to start the reaction on the metallic alloy.

Parallel to recording the H_2_ signal, we measured *operando* XP spectra of the Ga 3 d, Pt 4f, and C 1s core levels; see waterfall plots in Figure 1. Figure 2b shows the thickness of the GaO_x_ layer calculated from the Ga^0^ and Ga^3+^ components in the Ga 3d region in Figure 1a. Before the start of the experiment, we find a 0.8 Å oxide film that grows on the surface to 2.0 Å due to adsorption of oxygen‐containing residual gases in the chamber. After starting the heating ramp at t = 0.0 h, this oxide film becomes thinner, parallel to the onset of H_2_ evolution (0.2 h), and vanishes at ≈0.4 h. Shortly after reaching 800 K, the sample is oxide‐free. Thus, during the catalytic reaction, that is, between 1.3 and 3.3 h, no Ga^3+^ contribution was detected. This observation suggests that the reducing conditions and the high temperature during propene dehydrogenation result in the removal of the oxide. After 3.3 h, the system is cooled, and the H_2_ signal decreases. Initially, no changes of the Ga 3d signal are seen, but starting from 3.6 h (395 K), the regrowth of an oxide layer is detected to a thickness of 1.3 Å, due to adsorption of oxygen‐containing residual gases.

The Pt 4f spectra collected during the *operando* experiment are shown in Figure 1c as a waterfall plot. The Pt 4f_7/2_ binding energy obtained by peak fitting is plotted in Figure 2c. The signal obtained at 300 K before heating (from −0.3 to −0.1 h) gives a binding energy of 72.4 ± 0.1 eV. This Pt 4f_7/2_ peak position is assigned to Pt‐rich intermetallic phases, as discussed for Figure 1d. Upon heating, between 0.0 and 0.3 h, a new species with a lower Pt 4f_7/2_ binding energy emerges, which reaches a stable value of 71.9 ± 0.1 eV at ≈760 K, 0.3 h. We assign this binding energy to single Pt atoms in liquid Ga,^[^ ^11^, ^31^, ^46^ ^]^ due to the transition to a fully liquid GaPt alloy with 1.0 at.% Pt at ≈530 K.^[^ ^37^ ^]^ These single Pt atoms are present during the reaction at 800 K (1.3–3.3 h) and are assigned to the active site. Upon decreasing the temperature (>3.3 h), the Pt peak shifts back to reach 72.3 ± 0.1 eV at ≈620 K. We assign this shift to the reformation of Pt‐rich phases, similar to those present prior to the experiment.

The concentration of Pt at the surface is shown in Figure 2d. From the sample preparation, the nominal bulk concentration is known to be 1.0 at.% Pt. Prior to heating (from −0.3 to −0.1 h), we determine a concentration of 1.4 ± 0.2 at.%, indicating the formation of Pt‐rich phases at or near the surface. After heating the sample to 800 K (>0.3 h), the concentration decreases to 0.8 ± 0.1 at.% Pt. In literature, the occurrence of surface layering has been reported for GaPt liquid metal alloys, with Ga segregating to the surface due to a lower surface free energy.^[^ ^11^ ^]^ The resulting depletion of Pt from the surface is leading to a concentration slightly below the bulk concentration of 1.0 at.%. Note that the concentration of Pt on the outer surface is >0 at.% as determined by DFT^[^ ^11^ ^]^ calculations and shown in DRIFTS^[^ ^13^ ^]^ *operando* adsorption experiments. During the cooling of the sample (>3.3 h), Pt‐rich phases form again at the surface, and the Pt concentration goes up to 1.0 ± 0.2 at.%.

Figure 2e shows the evolution of the thickness of the carbon layer and the gas phase propane signal during the experiment. The gas phase signal remains constant throughout the experiment within the accuracy of the measurement (137 ± 47 cps; the low signal‐to‐noise ratio is due to fast acquisition in *operando* measurements). Prior to heating (300 K, from −0.3 to −0.1 h), a surface carbon signal corresponding to 0.6 monolayers (ML) carbon is observed. Upon heating to 800 K, the carbon coverage initially increases (0.9 ML at ≈0.6 h), and thereafter returns to ≈0.6 ML (corresponding to ≈0.2 ML C_3_ units per Ga atom on the surface) during the reaction (1.3–3.3 h). This coverage is significantly higher than the number of Pt sites and thus indicates that the carbon species is predominantly adsorbed on Ga, in line with previous calculations for the related GaRh system.^[^ ^17^ ^]^ Upon cooling and as the reaction stops (≈620 K, 3.4–3.5 h), the surface carbon species disappears, coinciding with the shift of the Pt species. This behavior suggests that we observe a volatile carbon reaction intermediate on the surface during the reaction, which is not formed below the reaction temperature, that is, below ≈620 K. Upon further cooling, a carbon layer, which is spectroscopically similar to the initially observed species, regrows on the surface following the oxide growth at lower temperatures (≈395 K, 4.3–4.4 h).

After retrieving the sample from the chamber, all metal and ceramic parts of the sample holder were covered by a coke layer, with the exception being the GaPt alloy surface (Figure S4, Supporting Information for photographs). This visualizes the reported superb coking resistance of the liquid metal catalyst, which is also confirmed by XPS herein and in previous studies.^[^ ^14^, ^23^ ^]^ Our data therefore convincingly suggest that the active catalyst is metallic and that dispersed Pt atoms form the active sites, while significant coking of the model SCALMS alloy is not observed.

### 2.2 Catalyst Deactivation by Oxygen

**Figure 3:**
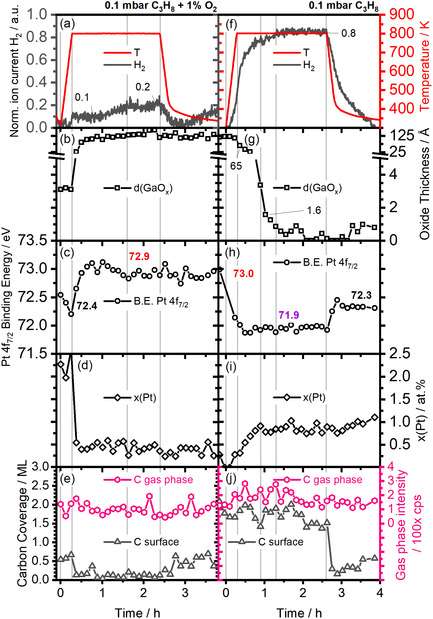
Operando data (0.1 mbar C_3_H_8_, 0.001 mbar O_2_) for a 1 at.% Pt in liquid Ga sample: a) activity, b) oxide thickness, c) Pt binding energy, d) Pt concentration in the surface near region, e) carbon coverage and gas phase signal (vertical lines at 0.0, 0.3, 1.6, 2.4 h). Operando data (0.1 mbar C_3_H_8_) of a fully oxidized GaPt droplet with 1 at.% Pt in the sample: f) activity, g) oxide thickness, h) Pt binding energy, i) Pt concentration in the surface near region, and j) carbon coverage and gas phase signal (vertical lines at 0.0, 0.3, 0.9, 1.3, and 2.6 h).

To evaluate the influence of oxygen in the feed stream on the activity of the GaPt catalyst, we exposed the sample to an additional pressure of 1 × 10^−3^ mbar O_2_ (≈1%) during PDH at ≈0.1 mbar C_3_H_8_. The corresponding results are shown in **Figure** 3. Figure 3a shows the H_2_ signal as a measure of the catalyst activity. The sample is again heated at a rate of 30 K min^−1^ starting at 0.0 h up to 800 K. When the reaction temperature is reached at 0.3 h, a small increase in H_2_ partial pressure to 0.1 a.u is observed, which slowly increases further after ≈1.0 h. Between 1.6 and 2.4 h, the pressure is roughly stable at 0.2 a.u. The comparison to the oxygen‐free situation in Figure 2a shows that the H_2_ signal is lower by a factor of 4. The comparison to the background activity of a blank sample holder and of a pure Ga droplet (without Pt) in 0.1 mbar C_3_H_8_ in Figure S5, Supporting Information, reveals that the thick Ga‐oxide film formed in the oxygen‐containing feed shows no significant activity. This is in line with the literature, where GaO_x_ catalysts^[^ ^47^ ^]^ show a significantly lower activity than SCALMS catalysts.^[^ ^11^ ^]^ When the sample is cooled to room temperature after 2.4 h, the H_2_ signal decreases to reach the background value at 3.0 h.

The analysis of the simultaneously recorded XP spectra allows us to follow the chemical state of the catalyst surface. Figure 3b shows the thickness of the oxide layer during the *operando* experiment (see Figure S6, Supporting Information for all spectra). Initially (0.0–0.3 h), a thin oxide of 3.1 ± 0.1 Å is present from reactions with residual gases. This observation is typical for oxophilic Ga, which passivates by forming a thin oxide layer even at low temperatures.^[^ ^31^, ^38^, ^41^ ^]^ At 800 K (>0.3 h), the rapid growth of a thick oxide film is observed due to increased diffusion, following the Cabrera–Mott model.^[^ ^48^, ^49^ ^]^ The film thickness reaches at least 90 Å after 0.5 h, which corresponds to the XPS information depth (Ga 3d, Al K_α_), that is, the highest thickness detectable in the experiment (higher thicknesses will not lead to an increase of the XP signal). The oxide film is stable and is also found on the sample surface after cooling the sample (t > 2.4 h).

Figure 3c shows the Pt 4f_7/2_ binding energy during the experiment (for the complete data see Figure S6d, Supporting Information). Initially (0.0–0.3 h), when only a thin oxide layer is present, the Pt 4f_7/2_ peak is found at 72.4 ± 0.2 eV, which is again assigned to the Pt‐rich phase detected in the previous experiment (without oxygen). After heating to 800 K (>0.3 h), the Pt 4f_7/2_ signal shifts by +0.5 eV to 72.9 ± 0.1 eV during oxide growth. This binding energy arises from Pt being incorporated in GaO_x_ or sitting on the oxide;^[^ ^31^, ^50^, ^51^ ^]^ it remains constant for the rest of the experiment.

The Pt concentration during the experiment is shown in Figure 3d. Initially (up to 0.3 h), it shows a value of 2.3 ± 0.3 at.% Pt. This surface enrichment reflects the previously reported affinity of Pt to the liquid‐oxide interface.^[^ ^38^ ^]^ After heating to 800 K, the Pt concentration drops, since the Ga alloy/GaO_x_ interface becomes more and more buried underneath the thickening oxide layer. It reaches 0.4 ± 0.1 at.% and stays constant during the rest of the experiment, what we interpret as some Pt being incorporated in the GaO_x_ or being on the GaO_x_ surface.^[^ ^31^ ^]^

Figure 3e shows the evolution of the thickness of the carbon layer and the gas phase propane C 1s signal during the experiment. The gas phase signal remains constant throughout the experiment (99 ± 39 cps). On the surface, initially (0.0 h), a carbon layer of 0.6 ML is observed. After heating to 800 K, between 0.4 and 2.4 h, only ≈0.1 ML remains, which returns to the initial 0.6 ML after cooling (>2.4 h). The disappearance of the carbon layer coincides with reaching 800 K and is interpreted as the removal of surface contaminations by reaction with oxygen. Notably, in the presence of oxygen, the amount of C surface species is significantly lower as compared to the experiment without oxygen in the feed stream (e.g., 0.6 ML of C in Figure 2).

These results show that when a significant oxygen pressure (≈1%) is present, an oxide layer forms that inhibits the catalytic activity of the SCALMS catalyst. The small remaining activity might be associated with the Pt at the GaO_x_ surface and/or the “blind” activity of the sample holder and chamber (see Figure S5, Supporting Information).

### 2.3 Catalyst Reactivation

After demonstrating that the catalyst is deactivated by oxygen, the next important question to be treated is the activation or the reactivation of the catalyst after oxygen treatment. Thereby, oxides formed by handling a catalyst in air or due to burning‐off of carbon deposits are removed to regain the active metallic catalyst. To model this treatment, we use the oxidized SCALMS droplet investigated above and expose it to propane at ≈0.1 mbar under reaction conditions. The corresponding results are shown in Figure 3f–j (for the XP spectra, see Figure S7, Supporting Information).

Figure 3f shows the H_2_ signal as a measure of the catalyst activity in a feed stream of ≈0.1 mbar propane with no oxygen. The experiment starts with the oxide film of at least 90 Å thickness (XPS information depth). The sample is again heated at a rate of 30 K min^−1^ to reach 800 K (at 0.3 h). The H_2_ partial pressure begins to rise at ≈500 K (0.1 h) and increases steadily to reach ≈0.8 a.u. after 0.9 h and a plateau after 1.3 h slightly higher at 0.8 a.u.; notably, in the initial experiment (Figure 1), this value was reached only at the end of the experiment (≈3.0 h). This observation suggests a significantly shorter activation period. Upon cooling with −30 K min^−1^, the activity, as deduced from the H_2_ partial pressure, drops to reach zero at ≈3.8 h.

Figure 3g shows the evolution of oxide thickness during the experiment, as deduced from the Ga 3d spectra (Figure S7, Supporting Information). The experiment starts with an oxide layer thicker than the information depth (≈90 Å); for this film, no metallic Ga is detected up to 0.4 h. As the sample temperature reaches 800 K, the oxide thickness starts to decrease. Interestingly, the presence of the oxide layer is not directly correlated with the reactivity, which reaches half of the maximum activity (0.4 a.u.) at 0.3 h at an oxide layer thickness of 65 Å. Considering the size of the unit cell of Ga_2_O_3_ of ≈6 Å, more than ten layers of GaO_x_ are still present, while the catalyst is already active.^[^ ^52^, ^53^ ^]^ The significant activity that is reached despite the oxide layer shows that an active phase is formed at the surface. This observation could suggest that the high activity is related to the surface reduction of the Pt‐doped GaO_x_ film, enabling the formation of catalytically active single Pt atoms in a liquid Ga matrix, as will be further discussed below. Please note that the film morphology could be inhomogeneous, such that parts of the oxide film have already been completely broken up, enabling direct access to the liquid GaPt alloy phase underneath. After 1.3 h, coinciding with the plateau and maximum H_2_ partial pressure (Figure 3f), the oxide is mostly gone (<1 Å). The oxide reappears when the sample is cooled down to room temperature (>3 h) due to adsorption of oxygen‐containing residual gas.

The Pt 4f_7/2_ binding energy is shown in Figure 3h and allows us to pinpoint the chemical state of the catalytically active Pt atoms during the reactivation of the catalyst. Initially, the binding energy is at 73.0 eV, typical for Pt incorporated in or in part on GaO_x_. At 0.3 h, as 800 K and 50% of the maximum activity is reached, the main contribution in the Pt 4f_7/2_ region has already shifted to 71.9 eV, although a 20% contribution at 72.9 eV is still present between 0.4 and 0.8 h (Figure S7e, Supporting Information); these observations indicate the coexistence of Pt in the oxide and a liquid metal alloy phase. The appearance of the single Pt atoms in a Ga matrix thus coincides with the rising high activity of the sample. The single Pt atoms with the Pf 4f_7/2_ level at 71.9 ± 0.1 eV become the only Pt species at 0.9 h and remain present during the reaction. Only upon cooling (>2.6 h) of the sample, the binding energy shifts to 72.3 ± 0.1 eV, which is attributed to the formation of the Pt‐rich phase at low temperatures (as was also observed in the initial experiment; see Figure 2c).

Figure 3i shows the concentration of Pt at the surface. Initially, the Pt concentration in the thick GaO_x_ film is 0.3 at.%, similar to the situation at the end of the last experiment with 1% O_2_ described in Figure 3a–e. Notably, at 0.0 and 0.1 h, the Pt concentration is below the detection limit. At 0.3 h and 800 K, we reach half of the maximum activity (H_2_ partial pressure) and half the nominal Pt concentration (≈0.4 at.%), demonstrating the correlation between the active site and the activity. The Pt concentration steadily increases until reaching a constant value of 0.8 ± 0.1 at.% at 0.9 h, which coincides with the first H_2_ signal plateau in Figure 2e and is expected for the active fully liquid alloy. The H_2_ signal continues to increase only slightly (+10%) as the remaining oxide is removed. This observation leads us to conclude that the majority of active sites are formed, even when there is still an oxidic phase present. After the cooling of the sample (>2.6 h), a slight increase in Pt concentration is observed as Pt‐rich phases form at the surface, matching the previous observations in Figure 2.

The evolution of the thickness of the carbon layer and the gas phase propane C 1s signal during the experiments is shown in Figure 3j. The gas phase signal remains constant throughout the experiment within our accuracy (172 ± 46 cps), as expected. For the surface signal, at 300 K (−0.2 h), a carbon layer of 2.1 ML is observed, which is significantly thicker than the carbon layers formed in the previous experiments; it is attributed to a larger degree of carbonaceous species from the background (5 days at 10^−9^ mbar). As the system turns active, the surface carbon thickness drops only slowly to reach 1.4 ML at 2.1 h. This is ≈2x higher than in the initial experiment, indicating the presence of additional carbon surface species. Notably, a pronounced drop in surface carbon intensity is observed upon cooling, at 2.5–2.7 h, as the dehydrogenation reaction stops and Pt form an intermetallic phase (same behavior as in Figure 2e, but here ≈0.2 ML remain, and 0 ML are not reached). Thereafter, a carbon layer regrows on the surface to 0.6 ML at 3.6 h.

Our investigations demonstrate the reductive activation or reactivation of the catalyst. Particularly interesting is the short activation period of the oxidized SCALMS model system, which suggests that Pt embedded in a GaO_x_ matrix is a highly reactive precursor and can thus lead to a new interesting synthetic path to more active SCALMS catalysts.

## 3 Conclusion

Our *operando* investigation of GaPt model SCALMS surfaces in the context of PDH using a NAPXPS chamber has led to first valuable spectroscopic insights into the behavior of these cutting‐edge catalyst materials under operation conditions. Our explorations of GaPt model SCALMS have yielded a more profound comprehension of the dynamic surface processes that drive their activity. The NAPXPS experiment enabled us to closely analyze surface reactions under relevant reaction conditions, mirroring the challenging environment of industrial PDH processes. Our findings reveal that the metallic and liquid GaPt surface is the most active phase, while oxidation of the surface poisons the catalyst. Because of the reductive conditions during dehydrogenation, the system can reduce to the metallic state, provided that no significant amount of oxygen is present in the feed. This reduction is facilitated by the incorporation of Pt in GaO_x_. Further, the SCALMS system showed superb coking resilience, as seen both by spectroscopy and visual inspection of the sample holder. We conclude that a liquid metal catalyst with perfect wetting properties on a porous support (SCALMS) could serve as an excellent catalyst for dehydrogenation without the risk of coking, significantly improving current catalytic processes.

Additionally, the insights obtained from this study open exciting avenues for further research and development in the field of catalysis. GaPt model SCALMS, with their unique liquid‐like properties and catalytically active single‐atom sites, hold promise for addressing crucial challenges in the industry and beyond.

## Supporting Information

Supporting Information is available from the Wiley Online Library or from the author.

## Conflict of Interest

The authors declare no conflict of interest.

## Supporting information

Supplementary Material

## Data Availability

Data is available at zenodo.org https://doi.org/10.5281/zenodo.15517610.
