# Supplementary material for: Operando Study of the Active Phase in Liquid GaPt Alloy Catalysts
Source: Small Sci. 2025 Nov 28;6(1):e202500423. doi: 10.1002/smsc.202500423 (PMC12798791; doi:10.1002/smsc.202500423)
Supplement: Supplementary file 1 — Supplementary Material [file SMSC-6-e202500423-s001.pdf]

Supporting Information for

Operando Study of the Active Phase in Liquid GaPt Alloy Catalysts

Michael S. Moritz[1,2], Christoph Wichmann[1,2], Marius Steinmetz[2], Hans-Peter Steinrück[1], and Christian Papp[2*]

[1] *Lehrstuhl für Physikalische Chemie II, Friedrich-Alexander-Universität Erlangen-Nürnberg, Egerlandstr. 3, 91058 Erlangen, Germany.*

[2] *Angewandte Physikalische Chemie, Freie Universität Berlin, Arnimallee 22, 14195 Berlin, Germany*

* christian.papp@fu-berlin.de

Experimental Section

Sample treatment

The model catalyst sample was prepared by weighing in respective amounts of Ga (99.9995%, Sigma Aldrich, ~1 g) and Pt (Heraeus). The alloy droplet is supported on a W boat (Kurt J. Lesker). The pristine metal alloy sample was cleaned by Ar^+ sputtering and annealed at >800 K in UHV. The sample temperature is measured by a K-type thermocouple spot welded to the bottom of the W boat containing the sample. The accuracy is estimated to be ± 2 K.[1]

The chamber was purged with $1x10^{-3}$ mbar propane (Air Liquide, 99.95%) before each measurement to minimize background impurities such as water during the *operando* measurements. During the measurements, propane was dosed directly onto the sample surface utilizing tubes directed towards the sample for beam dosing.[2,3] This setup leads to an effective pressure on the sample surface of ~0.1 mbar, at a background pressure of $1x10^{-3}$ mbar.[2,3] The corresponding oxides were formed *operando* by co-dosing $1x10^{-3}$ mbar O_2 (Linde, ≥ 99.5) into the chamber volume *via* background dosing.

NAPXPS and QMS

XPS was measured using a specialized laboratory instrument described in literature.[3] It is equipped with a non-monochromatized dual X-ray anode (SPECS XR 50 NAP), which was operated using the Al anode for X-ray generation (photon energy: 1486.6 eV). Photoelectrons were analyzed using a customized hemispherical analyzer (Omicron EA 125X U7). For gas phase analysis, the system is equipped with a quadrupole mass spectrometer (Pfeiffer Vacuum QMS 200 Prisma). Furthermore, a preparation chamber attached to the measurement chamber enables direct UHV transfer after Ar^+ sputtering.

XPS and QMS data evaluation

XPS data was evaluated using CasaXPS Version 2.3.18PR1.0. Signals were fitted using Gaussian-Lorentzian product functions (GL(70) as implemented in CasaXPS) with a Shirley background. The Ga 3d signals were fitted with a linear background and GL(70) functions for oxidic and GL(70)T(1) for metallic signals to account for the asymmetry.[4] Signals were referenced using the

Ga 3d region, as the binding energy was found to be independent of experimental conditions, as checked by the Fermi edge position. For quantification, sensitivity factors were calculated from the respective photoionization cross-section, the inelastic mean free path, and the inverse kinetic energy of the photoelectron.[5] The corresponding values were taken from Sessa V2.2.0.[6]

The Ga 3d region was investigated due to its frequent use in literature[1,7–10] and the higher kinetic energy (1466 eV vs 370 eV for Ga 2p with Al K_α) that leads to a decreased damping of the signal in the gas phase. Furthermore, the similar kinetic energy of the electrons from the Pt 4f region reduces the error in the quantification of the data.

H_2 was used as a metric for activity, as results for propane and propene signals are inconclusive due to the overlapping fragmentation pattern (Figure S1). The time-dependent QMS signal of m/z = 2 (H_2) was divided by a linear background (2 points, set at t = 0.0 h and after the cooling ramp) to normalize and compare the amount / partial pressure of H_2 released during the reactions.

Oxide layer thicknesses were calculated following the Hill method.[11,12] The signal intensities are corrected for the respective atom densities in the phases. The atoms per cubic Å in liquid Ga^0 is determined as 0.053 Ga/Å^3 and in Ga_2O_3 as 0.038 Ga/Å^3 from molecular weight and density.[13] Particularly for low oxide thicknesses, below 10 Å, see Figures S2, we expect an error of up to 25% resulting from the difficult fitting of the data, including systematic errors such as the IMFP.

By applying exponential attenuation (Lambert-Beer's law) with the inelastic mean free path (IMFP), we estimate the Ga 3d intensity of the first atom layer (2.7 Å) to be 8% of the overall Ga 3d intensity. From the relative peak areas of Ga 3d (× 0.08) and C 1s, we calculated the amount of C on the surface under the assumption that C does not dissolve in Ga. Throughout the paper, we will refer to the carbon coverage in monolayers (ML), that is, C atoms per surface Ga atom. Please note that we did not correct the coverage for the different unit cell of GaO_x.

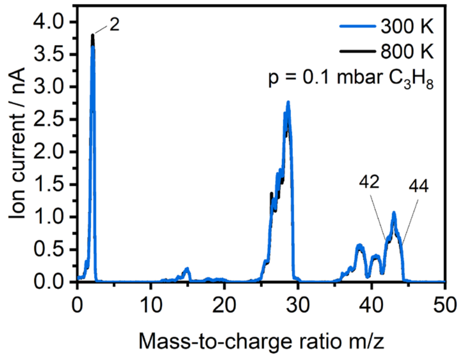

Figure S1: QMS at 300 and 800 K (reaction temperature) with 0.1 mbar C_3H_8 (m/z = 44) dosed onto a 1 at.% Pt in Ga surface: reaction products of propane dehydrogenation (PDH) are C_3H_6 (m/z = 42) and H_2 (m/z = 2); during ionization.

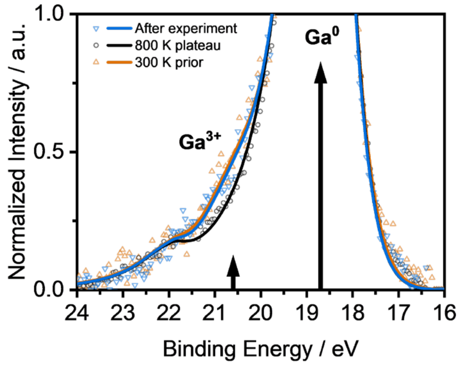

Figure S2: Zoomed Ga 3d spectra of Figure 1 to show the Ga^{3+} contribution at 20.6 eV before and after the experiment.

a) b)

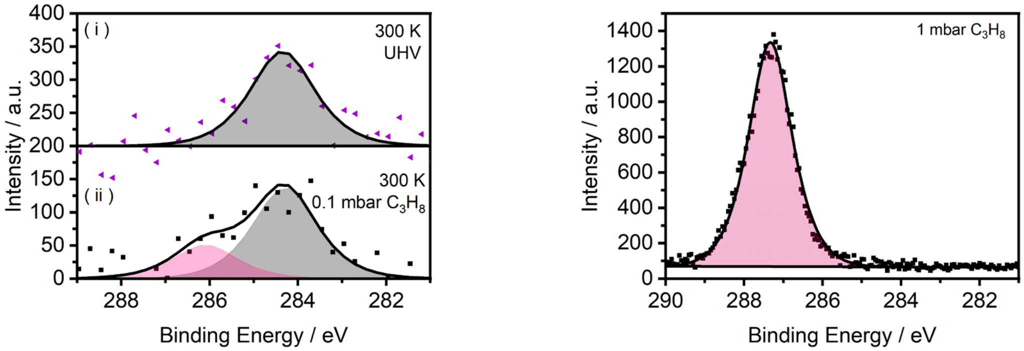

Figure S3: a) (i) C 1s region at 300 K in UHV, showing only the surface species and (ii) surface and gas phase signal at 0.1 mbar C_3H_8; b) shows the propane gas phase C 1s signal without any sample.

a) b) c)

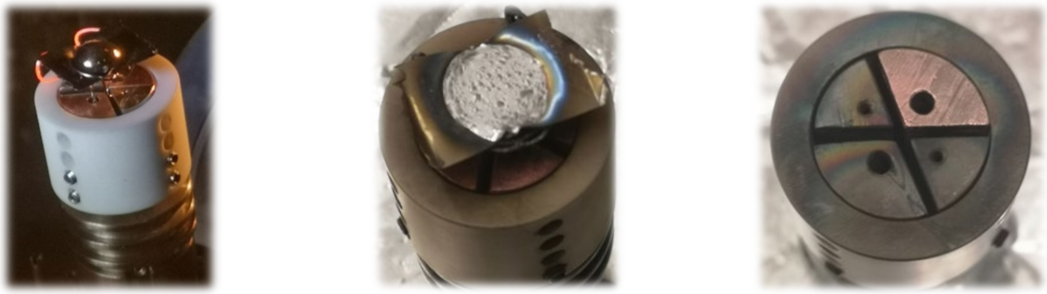

Figure S4: Sample holder a) before reaction; b) after reaction (ex-situ and therefore covered by oxide); c) after cleaning one Cu heating block for contrast, demonstrating the coking resistance of Ga-rich catalysts.

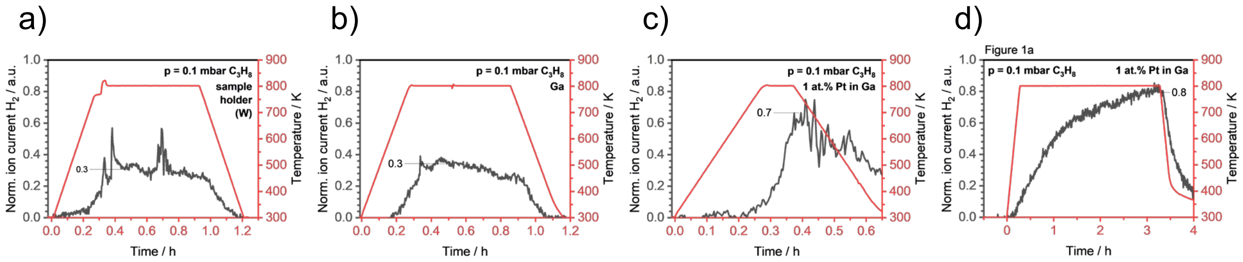

Figure S5: Reproduced activity data: a) only sample holder for background activity, b) only Ga, also showing background activity, c) a new 1 at.% Pt in Ga alloy, d) the GaPt alloy data of the main manuscript (Figure 1a (i)). t = 0 marks the start of the heating ramp.

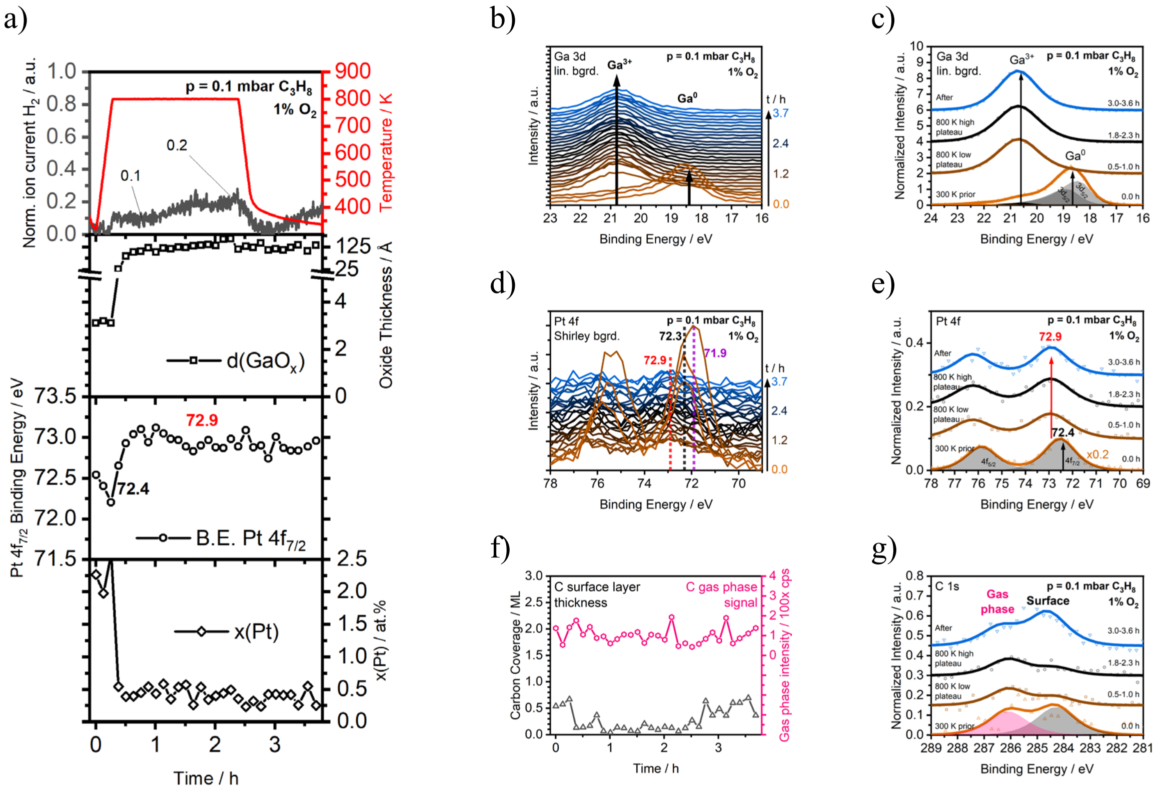

Figure S6: a) Operando data (0.1 mbar C₃H₈, 0.001 mbar O₂) for a 1 at% Pt in liquid Ga sample, from the top: activity, oxide thickness, Pt binding energy, and bottom the amount of active component; b) tr-XPS of the Ga 3d region; c) averaged Ga 3d spectra of relevant time frames (prior to heating: 0.0 h, initial activity at 800 K: 0.5-1.0 h, plateau at 800K: 1.8-2.3 h, after heating: 3.0-3.6 h) with fit envelope; d) tr-XPS of the Pt 4f region, and e) shows the averaged Pt 4f region for relevant time frames with fit envelope; f) carbon coverage; g) averaged C 1s region with fit envelope.

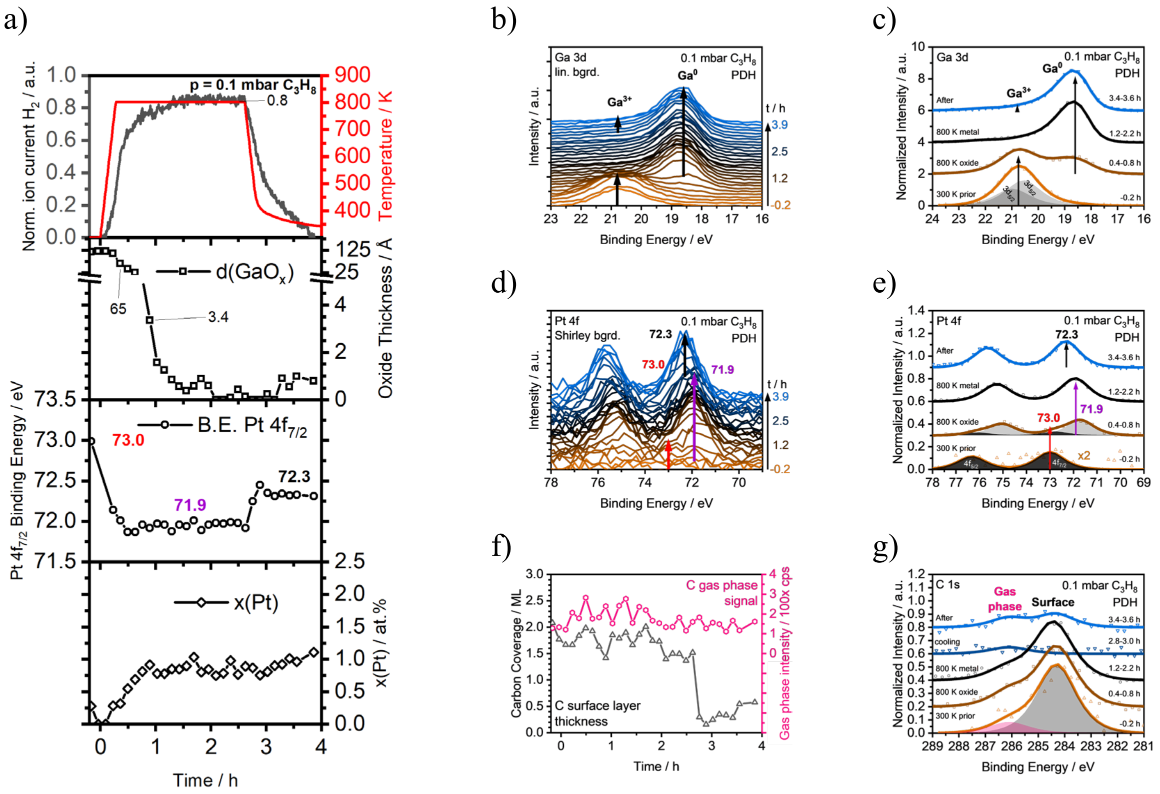

Figure S7: a) Operando data (0.1 mbar C_3H_8) of a fully oxidized GaPt droplet with 1 at% Pt in the sample, from the top: activity, oxide thickness, Pt binding energy, and bottom the amount of active component; b) tr-XPS of the Ga 3d region; c) averaged Ga 3d spectra of relevant time frames (prior to heating: -0.2 h, oxide traces at 800 K: 0.4-0.8 h, plateau at 800 K: 1.2-2.2 h, after heating: 3.4-3.6 h) with fit envelope; d) tr-XPS of the Pt 4f region, and e) shows the averaged Pt 4f region for relevant time frames with fit envelope; f) thickness of the carbon layer; g) averaged C 1s region with fit envelope and additional time frame as the surface carbon disappears during cooling (2.8-3.0 h).

Literature

[1] M. Grabau, S. Krick Calderón, F. Rietzler, I. Niedermaier, N. Taccardi, P. Wasserscheid, F. Maier, H. P. Steinrück, C. Papp, "Surface enrichment of Pt in Ga2O3 films grown on liquid Pt/Ga alloys" *Surf Sci* **2016**, *651*, 16–21.

[2] J. Pantförder, Photoelektronenspektroskopie Im "Pressure Gap" – Aufbau Einer Neuen Apparatur für Messungen im Druckbereich von 10^-10 bis 1 mbar, Friedrich-Alexander-Universität Erlangen-Nürnberg, **2005**.

[3] J. Pantförder, S. Pöllmann, J. F. Zhu, D. Borgmann, R. Denecke, H.-P. Steinrück, "New setup for *in situ* x-ray photoelectron spectroscopy from ultrahigh vacuum to 1mbar" *Rev Sci Instrum* **2005**, *76*, 014102.

[4] T. R. Gengenbach, G. H. Major, M. R. Linford, C. D. Easton, "Practical guides for x-ray photoelectron spectroscopy (XPS): Interpreting the carbon 1s spectrum" *J Vac Sci Technol A* **2021**, *39*, 013204.

[5] C. R. Brundle, B. V. Crist, "X-ray photoelectron spectroscopy: A perspective on quantitation accuracy for composition analysis of homogeneous materials" *J Vac Sci Technol A* **2020**, *38*, 041001.

[6] W. S. M. Werner, W. Smekal, C. J. Powell, *Simulation of Electron Spectra for Surface Analysis (SESSA) Version 2.2.0 User's Guide*, Gaithersburg, MD, **2021**.

[7] M. Dürrwächter, G. Indlekofer, H.-G. Boyen, P. Oelhafen, D. Quitmann, "Core level binding energy shifts in liquid binary alloys: Au-Ga" *J Non Cryst Solids* **1993**, *156–158*, 241–245.

[8] N. Raman, S. Maisel, M. Grabau, N. Taccardi, J. Debuschewitz, M. Wolf, H. Wittkämper, T. Bauer, M. Wu, M. Haumann, C. Papp, A. Görling, E. Spiecker, J. Libuda, H.-P. Steinrück, P. Wasserscheid, "Highly Effective Propane Dehydrogenation Using Ga–Rh Supported Catalytically Active Liquid Metal Solutions" *ACS Catal* **2019**, *9*, 9499–9507.

[9] N. Taccardi, M. Grabau, J. Debuschewitz, M. Distaso, M. Brandl, R. Hock, F. Maier, C. Papp, J. Erhard, C. Neiss, W. Peukert, A. Görling, H.-P. Steinrück, P. Wasserscheid,

"Gallium-rich Pd–Ga phases as supported liquid metal catalysts" *Nat Chem* **2017**, *9*, 862–867.

[10] G. Wowsnick, D. Teschner, I. Kasatkin, F. Girgsdies, M. Armbrüster, A. Zhang, Y. Grin, R. Schlögl, M. Behrens, "Surface dynamics of the intermetallic catalyst Pd2Ga, Part I – Structural stability in UHV and different gas atmospheres" *J Catal* **2014**, *309*, 209–220.

[11] J. M. Hill, D. G. Royce, C. S. Fadley, L. F. Wagner, F. J. Grunthaner, "Properties of oxidized silicon as determined by angular-dependent X-ray photoelectron spectroscopy" *Chem Phys Lett* **1976**, *44*, 225–231.

[12] P. J. Cumpson, "The Thickogram: a method for easy film thickness measurement in XPS" *Surf Interface Anal* **2000**, *29*, 403–406.

[13] H. Wittkämper, S. Maisel, M. Moritz, M. Grabau, A. Görling, H.-P. Steinrück, C. Papp, "Surface oxidation-induced restructuring of liquid Pd–Ga SCALMS model catalysts" *Phys Chem Chem Phys* **2021**, *23*, 16324–16333.
